# Generalization of fear-potentiated startle in the presence of auditory cues: a parametric analysis

**DOI:** 10.3389/fnbeh.2014.00361

**Published:** 2014-10-17

**Authors:** Seth Davin Norrholm, Tanja Jovanovic, Maria A. Briscione, Kemp M. Anderson, Cliffe K. Kwon, Victor T. Warren, Lauren Bosshardt, Bekh Bradley

**Affiliations:** ^1^Atlanta Veterans Affairs Medical Center, Mental Health Service LineDecatur, GA, USA; ^2^Department of Psychiatry and Behavioral Sciences, Emory University School of MedicineAtlanta, GA, USA

**Keywords:** startle response, generalization, stimulus, fear conditioning, auditory perception, translational medical research

## Abstract

Intense fear responses observed in trauma-, stressor-, and anxiety-related disorders can be elicited by a wide range of stimuli similar to those that were present during the traumatic event. The present study investigated the experimental utility of fear-potentiated startle paradigms to study this phenomenon, known as stimulus generalization, in healthy volunteers. Fear-potentiated startle refers to a relative increase in the acoustic startle response to a previously neutral stimulus that has been paired with an aversive stimulus. Specifically, in Experiment 1 an auditory pure tone (500 Hz) was used as the conditioned stimulus (CS+) and was reinforced with an unconditioned stimulus (US), an airblast to the larynx. A distinct tone (4000 Hz) was used as the nonreinforced stimulus (CS−) and was never paired with an airblast. Twenty-four hours later subjects underwent Re-training followed by a Generalization test, during which subjects were exposed to a range of generalization stimuli (GS) (250, 1000, 2000, 4000, 8000 Hz). In order to further examine the point at which fear no longer generalizes, a follow-up experiment (Experiment 2) was performed where a 4000 Hz pure tone was used as the CS+, and during the Generalization test, 2000 and 8000 Hz were used as GS. In both Experiment 1 and 2 there was significant discrimination in US expectancy responses on all stimuli during the Generalization Test, indicating the stimuli were perceptually distinct. In Experiment 1, participants showed similar levels of fear-potentiated startle to the GS that were adjacent to the CS+, and discriminated between stimuli that were 2 or more degrees from the CS+. Experiment 2 demonstrated no fear-potentiated startle generalization. The current study is the first to use auditory cues to test generalization of conditioned fear responses; such cues may be especially relevant to combat posttraumatic stress disorder (PTSD) where much of the traumatic exposure may involve sounds.

## Introduction

The fear-related symptoms of trauma-, stressor-, and anxiety-related disorders such as panic disorder, specific phobia, and posttraumatic stress disorder (PTSD) have been conceptualized within the framework of fear conditioning (Wolpe and Rowan, 1988; Friedman, 2000; Bouton et al., 2001; Mineka and Zinbarg, 2006; Norrholm and Jovanovic, 2010; Briscione et al., 2014) and empirical evidence suggests that these symptoms can arise as a result of impaired fear inhibition (Jovanovic et al., 2009, 2010, 2012; Jovanovic and Norrholm, 2011; Greenberg et al., 2013b) and/or over-generalization of fear responses (Lissek, 2012). From a clinical perspective, intense fear responses can be elicited by a wide range of stimuli that possess qualities similar to cues present during the index traumatic experience (Ehlers and Clark, 2000; Feldner et al., 2007), a phenomenon termed stimulus generalization (for review see Lissek, 2012).

In the laboratory, stimulus generalization methods involve the analysis of generalization gradients that are characterized by the weakening of conditioned fear responses as the perceptual similarity of the test stimuli and originally reinforced conditioned stimulus (CS) diminishes (Pavlov, 1927; Armony et al., 1997). Thus, in a typical generalization gradient, the strongest fear response occurs in response to the reinforced CS+ with a gradual reduction in conditioned fear responses as the test stimuli are perceived as increasingly different from the CS+. A steeper slope of the generalization gradient indicates less generalization whereas a shallower slope indicates increased generalization; the latter type of gradient has been suggested as a potential marker of pathologic anxiety (Keane et al., 1985; Foa et al., 1989; Grillon and Morgan, 1999; Lissek et al., 2008, 2010, 2014; Greenberg et al., 2013b). Ethologically speaking, generalization of fear responses to similar stimuli can be adaptive, however, excessive fear generalization may lead to maladaptive behaviors and the potential for psychopathology. Clinically, this may manifest in combat veterans with PTSD who experience uncontrollable fear when they see or hear a fearful cue (e.g., trash on the road or fireworks) even after they have returned home (Hoge, 2010).

For the latter part of the past four decades, examinations of stimulus generalization were conducted in a relatively large body of animal work (for an extensive review see Ghirlanda and Enquist, 2003) and much of this work was performed using appetitive conditioning procedures (e.g., Guttman and Kalish, 1956). An additional commonly employed translational, psychophysiological tool is fear-potentiation of the acoustic startle reflex. Fear-potentiated startle is defined as the increase in the frequency or intensity of the acoustic startle reflex in the presence of a previously neutral cue (termed a conditioned stimulus or CS; e.g., colored geometric shape) that has been associated with an aversive, unconditioned stimulus (US) (termed the US; e.g., cutaneous electric shock or airblast to the larynx; Davis and Astrachan, 1978; Davis et al., 1993, 1999). The integration of fear-potentiated startle measures and stimulus generalization techniques has only recently been investigated in psychiatrically healthy humans (Lissek et al., 2008) and populations presenting with anxiety disorder symptoms (Lissek et al., 2010). More specifically, Lissek et al. (2008) generated generalization gradients in humans using concentric circles of gradually changing diameter as generalization stimuli (GSs) and then later employed this paradigm to show over-generalization of fear in patients with panic disorder (Lissek et al., 2010) and generalized anxiety disorder (GAD; Lissek et al., 2014).

More recent work in fear generalization has used neuroimaging methods to examine specific brain networks underlying this phenomenon. For example, using functional magnetic resonance imaging (fMRI) and skin conductance response measures, Dunsmoor et al. (2011) generated a generalization gradient along a continuum of fearful-to-neutral faces. In that study, generalization of fear responses occurred in the presence of faces one unit of differentiation removed from the previously reinforced CS+ and this heightened fear response was correlated with neural activity in the amygdala, striatum, insula, thalamus, and periaqueductal gray (Dunsmoor et al., 2011); brain regions that are critical for the expression of learned fear as well as for adaptation to changes in CS-US contingencies (LeDoux et al., 1988; LeDoux, 1996; Berns et al., 2006; Dunsmoor et al., 2007; Delgado et al., 2008; Schiller and Delgado, 2010). In another similar set of experiments using faces as conditioned stimuli, Glenn and colleagues developed a paradigm that gradually morphed one individual into a different individual, both of which had an emotionally neutral expression (Glenn et al., 2012). One of the faces was the CS+ and the second face was the CS-, and generalization was tested using the morphed faces along the continuum. The US in these studies was the sound of a woman’s scream paired with the fearful expression of the CS face. The paradigm used fear-potentiated startle (Glenn et al., 2012) and fMRI (Britton et al., 2013) outcomes, in adolescents and adults with anxiety. These studies found that fear generalization was age dependent and disrupted by anxiety.

Greenberg et al. (2013b) employed a previously validated generalization paradigm using geometric shapes of varying sizes (see Greenberg et al., 2013a) to show less differential responding in patients with GAD (Greenberg et al., 2013b). These reported effects were associated with decreased activity in the ventromedial prefrontal cortex (vmPFC), a region associated with fear inhibition, and somatosensory areas (i.e., less steep neural gradients in response to generalization stimulus (GS) presentations of increasing dissimilarity, (Greenberg et al., 2013b). By comparison, psychiatrically healthy controls showed increased vmPFC activity as GSs became more distinct from the original reinforced CS+.

Within the field of visual stimuli, generalization has been tested along the hierarchy of bottom-up processing, from perceptual features to categorical representations. Specifically, generalization studies have employed different color wavelengths (Dunsmoor and LaBar, 2013), in addition to complex stimuli defined within categories of objects (e.g., animate vs. inanimate objects, (Dunsmoor et al., 2013) or even abstract, arbitrarily defined categories (Vervoort et al., 2014). Taken together, these studies suggest the fear generalization can extend from specific exemplars to broad categories and thus has significant implications for better understanding maladaptive behaviors related to stress and anxiety.

While there is a rich literature surrounding stimulus generalization, the impact of this phenomenon within other perceptual modalities, such as olfactory or auditory processing, is one area that remains largely understudied. Bremner et al. (1999) characterized the profound nature of auditory cues to elicit behavioral and neurological changes in Vietnam Veterans with PTSD. However, despite a surge of service members returning home with PTSD, the impact of auditory cues on stimulus generalization and subsequent PTSD symptomology has not been well studied in the context of PTSD symptomology (Bremner et al., 1999).

Thus, the purpose of the present study was to further investigate the experimental utility of a fear-potentiated startle-based stimulus generalization paradigm for potential use in clinical populations diagnosed with fear and anxiety disorders (e.g., van Meurs et al., 2014). The CSs employed in the current study were auditory pure tones selected from within the octave range that represents the spectrum associated with human communication and common ambient auditory stimuli (Arlinger, 1991; American Speech-Language-Hearing Association (ASHA), 2005) and these CSs are consistent with those used in our previous work with auditory fear conditioning stimuli (Norrholm et al., 2011). The rationale for furthering the study of stimulus generalization by incorporating auditory stimuli is based on several recent experimental and clinical findings including (1) empirical evidence showing robust fear conditioning to cues of this modality (Norrholm et al., 2011); (2) greater consistency with the basic animal work upon which translational human studies are based; and (3) the prevalence of traumatic events in PTSD populations that involved auditory elements (National Council on Disability, 2009).

The present study included two experiments: in the first one we tested pure tones of frequencies that differed from the CS+ from 250 Hz to 7500 Hz. In the second experiment we sought to examine generalization spanning one degree of separation from the CS+ based on frequency differences to which fear did not generalize in the first experiment. The goal of the second study was to determine whether generalization was driven by CS+/GS adjacency or difference in tone frequency.

## Materials and methods

### Participants

Seventy-seven participants (38 males/39 females) with a mean age of 25 ± 0.77 years were enrolled in this study after signing an informed consent form approved by the Emory University Institutional Review Board, the Atlanta VAMC Research and Development Committee, and the US Army Medical Research and Materiel Command (USAMRMC)/Office of Research Protections (ORP)/Human Research Protection Office (HRPO). The psychiatrically healthy volunteers included in this study were recruited as part of a larger investigation of fear inhibition and generalization in combat veterans at the Atlanta VAMC.

### Trial definitions

The eyeblink component of the acoustic startle response was measured according to previously published methods (Norrholm et al., 2006, 2011). Acoustic startle response magnitude was recorded via electromyography (EMG) readings of the right *orbicularis oculi* muscle. Two 5 mm Ag/AgCl electrodes filled with electrolyte gel were placed 1 cm below the pupil and 1 cm below the lateral canthus. EMG signals were amplified and digitalized with the BIOPAC MP150 monitoring system (Biopac Systems, Inc., Aero Camino, CA). Impedances through these electrodes were less than 6 kΩ. Startle magnitude was determined as the peak amplitude of the EMG contraction 20–250 ms following the acoustic stimulus.

The startle probe was a 108-dB [A], 40 ms burst of white noise with near instantaneous rise time delivered binaurally with headphones. Similar to several of our previous studies (e.g., Jovanovic et al., 2005; Norrholm et al., 2008), the aversive stimulus (US) was a 250 ms, 140 p.s.i. airblast directed at the larynx. The CSs (Fear Acquisition and Re-training) and GSs (Generalization Test) were auditory pure tones created using Adobe Audition for Windows, version 3.0 and were matched for perceptual loudness according to the principles described by Fletcher and Munson (1993) and in our previous work (Norrholm et al., 2011). CSs and GSs were presented to the participants for 6 s via the same binaural headphones used to deliver the startle probes. The CS+ and CS- were 500 Hz and 4000 Hz pure tones and the GSs spanned the octave range used in pure tone threshold audiometry (250, 1000, 2000, 4000, 8000 Hz). To prevent rapid habituation to the acoustic startle probe (due to the repeated auditory stimulation via acoustic startle probes and auditory CSs), a startle probe was included in only two of the four presentations of the CSs in each block during Fear Acquisition. All CS+ presentations during this phase were paired with the airblast US for 100% reinforcement schedule, regardless of whether or not an acoustic startle probe was included. On CS+ trials with an acoustic startle probe (during Fear Acquisition and Re-training), the tone was presented for 6 s total, with the 40 ms startle probe presented 5210 ms after CS onset followed 500 ms later by the 250 ms, 140 p.s.i. airblast that co-terminated with CS presentation. On CS+ trials without an acoustic startle probe (during Fear Acquisition), the tone was presented for 6 s total, for 5750 ms alone and then together the 250 ms, 140 p.s.i. airblast that co-terminated with CS presentation. On CS- trials (Fear Acquisition and Re-training) and GS trials (Generalization Test), the tone was presented for 6 s total, with the startle probe occurring 5960 ms after CS onset. On noise alone (NA) trials, the 40 ms startle probe was presented alone without the CSs.

### US expectancy

A three-button response keypad (SuperLab, Cedrus Corporation, San Pedro, CA) was used during each acoustic startle session to record the expectancy of the participants of the US on each CS presentation. Participants received verbal instructions prior to each session on how to respond with the keypad. Participants were instructed to press a button marked “+” if they expected the shape to be followed by the US, a button marked “−” if they did not expect the airblast US, or a button marked “0” if they were uncertain. Instructions were to press the button within 3 s of CS onset. Any responses occurring during or after the airblast US were discarded.

### Session definitions

#### Experiment 1

Forty-three individuals participated in Experiment 1. The experimental procedures occurred over the course of two consecutive days. The Fear Acquisition session occurred on Day 1 and the Re-training and Generalization Test occurred on Day 2. All test sessions occurred in the same context. The Fear Acquisition session began with a 1-min acclimation period followed by a habituation phase consisting of three NA presentations. Next, a CS habituation phase was presented consisting of two presentations of each CS without the airblast US. After habituation to the CSs, the Fear Acquisition session continued with three blocks of four trials of each trial type (CS+, CS-, NA). The inter-trial interval (ITI) was randomized between 9 and 22 s.

The Re-training and Generalization Test sessions occurred 24 h after Fear Acquisition. The Re-training phase began with a habituation phase consisting of three NA presentations. Following this brief habituation to the startle probe, the Re-training phase was administered and consisted of six NA trials, two reinforced CS+, and two non-reinforced CS− trials that were presented in a quasi-random order. Re-training was immediately followed by the Generalization Test that consisted of three trials each of the previously reinforced CS+, the non-reinforced CS-, and four GSs (250, 1000, 2000, and 8000 Hz). The units of differentiation used in the current study were octave intervals commonly used in pure tone threshold audiometry, given that these are considered perceptually different (Arlinger, 1991; American Speech-Language-Hearing Association (ASHA), 2005) and are considered the “gold standard” for assessing peripheral auditory function (Walker et al., 2013).

#### Experiment 2

Thirty four individuals participated in Experiment 2. The methods for the Fear Acquisition (Day 1) and Re-training (beginning of Day 2) sessions in the follow-up Experiment 2 were nearly identical to that of Experiment 1. The only difference was that the CS+ was a 4000 Hz pure tone and the CS- was 500 Hz for Experiment 2. The purpose of follow-up Experiment 2 was to increase the difference between the CS+ and the two adjacent GSs in an effort to better understand the point at which fear no longer generalizes. For this follow-up experiment, the octave structure of the pure tone GS was maintained such that octaves represented the units of differentiation. The abbreviated Generalization Test in Experiment 2 consisted of three trials each of the previously reinforced CS+ and two adjacent GSs (2000 and 8000 Hz).

## Results

### Experiment 1

#### Fear acquisition: fear-potentiated startle

**Figure 1:**
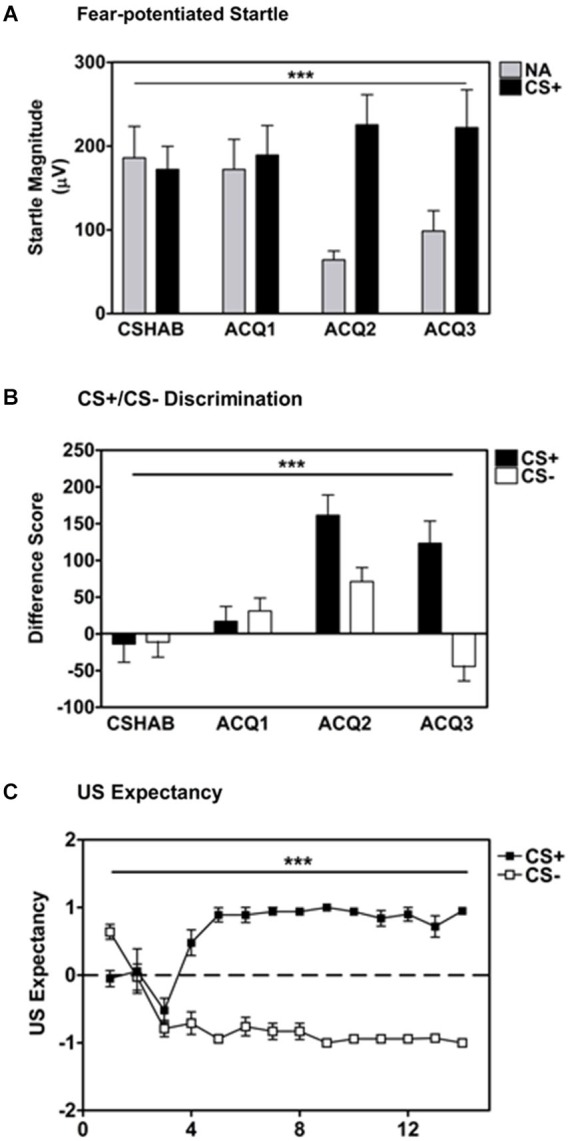
**Participants displayed robust fear-potentiated startle to the CS+ as compared to the noise probe alone (NA; panel A) and discrete discrimination between the CS+ and CS− based on fear-potentiated startle responses (panel B) and US expectancy ratings (panel C)**. Difference Score = [startle magnitude to the CS] − [startle magnitude to the noise probe alone]. *** *p* ≤ 0.001.

Participants developed robust fear-potentiated startle to the reinforced CS+ (500 Hz pure tone) as compared to startle responses to the noise probe alone (NA) across blocks, Repeated Measures ANOVA, Significant Block × Trial Type interaction, *F*_(3,126)_ = 10.67, *p* < 0.001, see Figure 1A. Startle magnitude in the last block of acquisition was much greater to the CS+ than NA, main effect of Trial Type, *F*_(1,42)_ = 16.82, *p* < 0.001.

#### Fear acquisition: CS+/CS- discrimination

Participants also developed significant discrimination between the reinforced CS+ (500 Hz pure tone) and the non-reinforced CS- (4000 Hz pure tone) across conditioning blocks, Repeated Measures ANOVA, Significant Block × Trial Type interaction, *F*_(3,126)_ = 14.75, *p* < 0.001, see Figure 1B. Again, the difference score (CS minus NA) for the CS+ was much greater than the CS- in the last block of conditioning, main effect of Trial Type *F*_(1,42)_ = 21.60, *p* < 0.001.

#### Fear acquisition: US expectancy

Based on US expectancy ratings, participants clearly discriminated between the CS+ and CS- during the Fear Acquisition session (Repeated Measures ANOVA, significant Trial × Trial Type interaction, *F*_(13,546)_ = 10.34, *p* < 0.001, see Figure 1C). These findings were confirmed during exit interviews with participants at the conclusion of the experimental session.

#### Re-training: CS+/CS- discrimination

**Figure 2:**
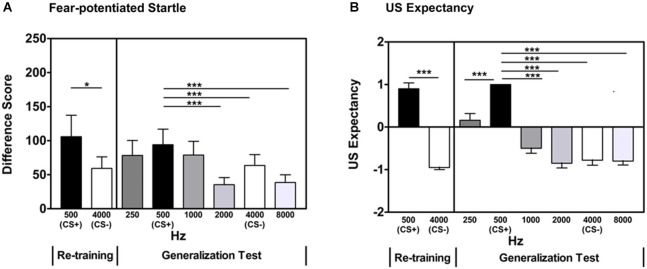
**In Experiment 1, participants displayed significant discrimination between the CS+ and CS- during the Re-training phase as measured by fear-potentiated startle (panel A) and US expectancy (panel B)**. A generalization gradient was evident when examining fear-potentiated startle to the previously reinforced CS+ and the GS two steps away from the CS+ (2000 Hz). There was no significant difference between the previously reinforced CS+ and the two adjacent GSs (250 and 1000 Hz). Based on US expectancy ratings, participants displayed clear retention of the excitatory properties of the previously reinforced CS+ and a generalization gradient was evident when examining expectancy ratings from the previously reinforced CS+ and the GS two steps away from the CS+ (2000 Hz). Difference Score = [startle magnitude to the CS] − [startle magnitude to the noise probe alone]. *p< 0.05; ***p ≤ 0.001.

After a brief acquisition Re-training phase, participants showed significant discrimination between the reinforced CS+ and non-reinforced CS- based on fear-potentiated startle measures (Repeated Measures ANOVA, significant main effect of Trial Type, *F*_(1,42)_ = 4.30, *p* = 0.04, see Figure 2A), as well as US expectancy measures (Repeated Measures ANOVA, significant main effect of Trial Type, *F*_(1,42)_ = 129.4, *p* < 0.001, Figure 2B).

#### Generalization: CS/GS discrimination

During the Stimulus Generalization Test, the previously used CS+ (500 Hz pure tone) and CS- (4000 Hz pure tone) were presented in a quasi-random sequence along with four GSs spanning 250–8000 Hz at octave intervals (three presentations each of 250, 500, 1000, 2000, 4000, and 8000 Hz). Repeated Measures ANOVA revealed a Main Effect of Stimulus (*F*_(5,210)_ = 7.47, *p* < 0.001). This was followed by contrast comparisons of each Stimulus frequency and the previously reinforced CS+ (500 Hz). There was no significant difference in the fear-potentiated startle response to the GSs that were closest (i.e., one unit of differentiation) to the 500 Hz CS+ (500 Hz CS+ vs. 250 Hz GS, *F*_(1,42)_ = 2.44, *p* = 0.13; 500 Hz CS+ vs. 1000 Hz GS, *F*_(1,42)_ = 1.48, 0.23). Fear-potentiated startle responses to the GSs that were more distal (i.e., greater than one unit of differentiation) from the 500 Hz CS+ were significantly different (500 Hz CS+ vs. 2000 Hz GS, *F*_(1,42)_ = 12.35, *p* = 0.001; 500 Hz CS+ vs. 4000 Hz CS-, *F*_(1,42)_ = 9.04, *p* = 0.004; 500 Hz CS+ vs. 8000 Hz GS, *F*_(1,42)_ = 15.0, *p* < 0.001). These differences remained significant when controlling for multiple comparisons (alpha level adjusted to 0.01 to account for number of comparisons). Table 1 lists the significance of the tests comparing the GS to the CS+ sorted by relative frequency difference between the two across both experiments. These data indicate that GSs that were one unit of differentiation removed from the CS+ were not significantly different, whereas those that were ≥2 units removed were significantly different from the CS+.

**Table 1:** **Significance of the Generalization Test trials comparing the GS to the CS+ on fear-potentiated startle and US expectancy measures**.

Difference from CS+ (Hz)	Fear-potentiated Startle	US expecatancy
250^1^	ns	*p* < 0.001
500^1^	ns	*p* < 0.001
1500^1^	*p* = 0.001	*p* < 0.001
2000^2^	*p* = 0.02	*p* < 0.001
3500^1^	*p* = 0.004	*p* < 0.001
4000^2^	*p* = 0.003	*p* < 0.001
7500^1^	*p* < 0.001	*p* < 0.001

*The tests are sorted by relative frequency difference between the two across both experiments*.

*^1^Trial Types from Experiment 1*.

*^2^Trial Types from Experiment 2*.

#### Generalization: US expectancy

Examination of US expectancy measures in the generalization test previously described revealed a Main Effect of Stimulus (Repeated Measures ANOVA *F*_(5,210)_ = 46.3, *p* < 0.001). This was followed by contrasts comparing each Stimulus frequency and the CS+ (500 Hz). US expectancy ratings on the CS+ trials compared to the CS- (4000 Hz; *F*_(1,42)_ = 133.6, *p* < 0.001) trials and all of the GS trials (250, 1000, 2000, 8000 Hz) were significantly different (500 Hz CS+ vs. 250 Hz GS, *F*_(1,42)_ = 24.6, *p* < 0.001; 500 Hz CS+ vs. 1000 Hz GS, *F*_(1,42)_ = 84.1, *p* < 0.001; 500 Hz CS+ vs. 2000 Hz GS, *F*_(1,42)_ = 190.0, *p* < 0.001; 500 Hz CS+ vs. 8000 Hz GS, *F*_(1,42)_ = 190.4, *p* < 0.001, see Table 1). These differences remained significant when controlling for multiple comparisons (alpha level adjusted to 0.01 to account for number of comparisons). These data indicate that the GSs were perceptually distinct from the CS+.

### Experiment 2

#### Fear acquisition: fear-potentiated startle, CS+/CS- discrimination, and US expectancy

In the follow-up experiment, a separate sample of psychiatrically healthy participants (*n* = 34) underwent a Fear Acquisition protocol identical to that which was performed in Experiment 1, however, the CS+ was a 4000 Hz pure tone and the CS- was a 500 Hz pure tone (counterbalanced from Experiment 1). Similar to Experiment 1, all participants displayed robust fear-potentiated startle to the CS+ as compared to NA (Repeated Measures ANOVA, significant Block × Trial Type interaction, *F*_(3,99)_ = 27.84, *p* < 0.001, main effect of Trial Type in the last block, *F*_(1,33)_ = 30.11, *p* < 0.001, significant discrimination between the CS+ and CS- (Repeated Measures ANOVA, significant Block × Trial Type interaction, *F*_(3,99)_ = 19.99, *p* < 0.001, main effect of Trial Type in the last block, *F*_(1,33)_ = 36.01, *p* < 0.001, and clear discrimination between the CS+ and CS- on US expectancy measures (Repeated Measures ANOVA, significant Trial × Trial Type interaction, *F*_(13,429)_ = 10.33, *p* < 0.001, main effect of Trial Type, *F*_(1,33)_ = 89.16, *p* < 0.001). There were no significant differences between the groups in Experiments 1 and 2 with regard to fear-potentiated startle (Repeated Measures ANOVA, no significant Block × Trial Type × Group interaction, *F*_(1,75)_ = 0.022, *p* = 0.88), CS+/CS- discrimination (Repeated Measures ANOVA, no significant Block × Trial Type × Group interaction, *F*_(1,75)_ = 0.004, *p* = 0.95), or US expectancy (Repeated Measures ANOVA, no significant Trial × Trial Type × Group interaction, *F*_(1,33)_ = 1.68, *p* = 0.21).

#### Re-training: CS+/CS- discrimination

**Figure 3:**
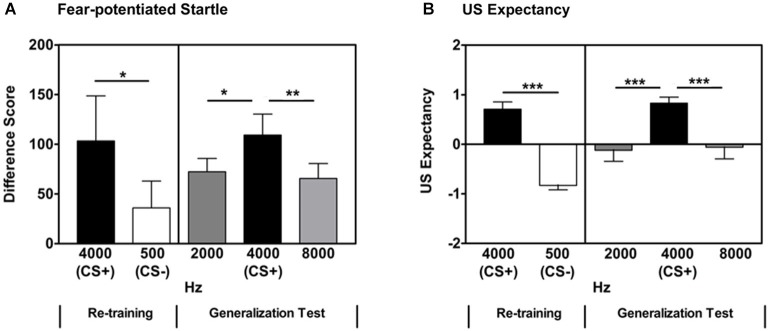
**Follow-up Experiment 2 was aimed at better understanding the point at which generalization occurs in this auditory stimulus generalization paradigm**. When the GSs differed from the previously reinforced CS+ by 2000 and 4000 Hz (maintaining octave intervals), there was a significant difference between the previously reinforced CS+ and the two GSs on both fear-potentiated startle responses (panel **A**) and US expectancy (panel **B**). Difference Score = [startle magnitude to the CS] − [startle magnitude to the noise probe alone]. * *p* < 0.05; ** *p* < 0.01; *** *p* ≤ 0.001.

After a brief acquisition Re-training phase, participants showed significant discrimination between the reinforced CS+ and non-reinforced CS- based on fear-potentiated startle measures (Repeated Measures ANOVA, significant main effect of Trial Type, *F*_(1,33)_ = 4.95, *p* = 0.03, see Figure 3A). There was no significant difference between the groups in Experiments 1 and 2 with regard to CS+/CS- discrimination, as measured by fear-potentiated startle, during Re-training (Repeated Measures ANOVA, no significant Trial Type × Group interaction, *F*_(1,75)_ = 0.31, *p* = 0.58).

#### Re-training: US expectancy

Participants also showed significant discrimination between the reinforced CS+ and non-reinforced CS- on US expectancy measures (Repeated Measures ANOVA, significant main effect of Trial Type, *F*_(1,33)_ = 77.3, *p* < 0.001, Figure 3B). There was no significant difference between the groups in Experiments 1 and 2 with regard to CS+/CS- discrimination, as measured by US Expectancy, during Re-training (Repeated Measures ANOVA, no significant Trial Type × Group interaction, *F*_(1,35)_ = 1.81, *p* = 0.19).

#### Generalization: GS/CS discrimination

In Experiment 1, using octave intervals, the CS+ (500 Hz) and the two adjacent GSs (250 and 1000 Hz) were separated by 250 and 500 Hz, respectively. During the Generalization test, there was no significant difference in fear-potentiated startle levels between the previously reinforced CS+ and these adjacent GSs. In order to examine the effect of frequency interval on stimulus generalization, a follow-up experiment was performed using the higher frequencies along the employed octave intervals (maintaining octaves as the units of differentiation). In the follow-up experiment (Experiment 2), a 4000 Hz pure tone was used as the CS+ for the Fear Acquisition phase. During the follow-up Generalization test, GSs of 2000 and 8000 Hz were administered. These differed from the CS+ by 2000 and 4000 Hz, respectively. With this frequency interval between the CS+ and GSs, the two adjacent GSs were significantly different from the previously reinforced CS+ (Repeated Measures ANOVA, significant main effect of Stimulus, *F*_(2,66)_ = 5.20, *p* = 0.008). This was followed by contrast comparisons of each adjacent Stimulus frequency (2000 and 8000 Hz) and the CS+ (4000 Hz; 4000 Hz CS+ vs. 2000 Hz GS, *F*_(1,33)_ = 6.0, *p* = 0.02; 4000 Hz CS+ vs. 8000 Hz GS, *F*_(1,33)_ = 10.0, *p* = 0.003, Figure 3A and Table 1). These differences remained significant when controlling for multiple comparisons (alpha level adjusted to 0.025 to account for number of comparisons).

#### Generalization: US expectancy

With the larger frequency intervals between the CS+ and GSs, similar to the fear-potentiated startle responses, the two adjacent GSs were significantly different from the previously reinforced CS+ (Repeated Measures ANOVA, significant main effect of Stimulus, *F*_(2,66)_ = 6.46, *p* = 0.004). This was followed by comparisons of each adjacent Stimulus frequency (2000 and 8000 Hz) and the previously reinforced CS+ (4000 Hz; 4000 Hz CS+ vs. 2000 Hz GS, *F*_(1,33)_ = 11.81, *p* = 0.003; 4000 Hz CS+ vs. 8000 Hz GS, *F*_(1,33)_ = 6.83, *p* = 0.02, Figure 3B and Table 1). These differences remained significant when controlling for multiple comparisons (alpha level adjusted to 0.025 to account for number of comparisons).

## Discussion

The primary findings of the current study were: (1) participants showed robust fear conditioning to the reinforced CS+ and significant discrimination between the auditory CS+ and CS- during Fear Acquisition, according to both fear-potentiated startle and US expectancy measures in a manner that replicated our previous work (Norrholm et al., 2011); (2) participants expressed fear to the GSs (250 and 1000 Hz) that were adjacent (i.e., one octave removed) to the previously reinforced CS+ (500 Hz), but not on GSs that were 2 or more degrees removed from the CS+ (Table 1), as assessed by fear-potentiated startle; (3) a follow-up experiment utilizing GSs (2000 and 8000 Hz) that were of greater frequency difference from the previously reinforced CS+ (4000 Hz) demonstrated a steeper gradient (less fear generalization) than that observed in the initial experiment; and (4) there was a dissociation between fear-potentiated startle responses and US expectancy responses during the Generalization Test. To our knowledge, in light of several emerging findings from the literature in the area of fear generalization in humans, this is the first to employ auditory cues as conditioning and GS.

In Experiment 1 of the current study, generalization of fear extended to the two GSs (250 and 1000 Hz) that were closest to the previously reinforced CS+ (500 Hz) and we observed the absence of generalization to the GSs that were farther from the CS+ (2000–8000 Hz). This is consistent with work by Lissek et al. (2008) who demonstrated fear generalization to visual cues that were one unit of differentiation (i.e., most similar) from the CS+. Of note, Lissek and colleagues used the CS- as the reference point for generalization, i.e., the GS trials that were significantly *different* from the CS- were considered to be generalization of CS+; in our study we directly compared the GSs to the previously reinforced CS+ to which the conditioned fear was originally acquired (see Vervoort et al., 2014) and essentially found the same effect. Of note, the participants did not show generalization between the CS+ and adjacent GSs on the US expectancy measure indicating that the stimuli were perceptually distinguishable from the CS+.

A follow-up experiment was conducted using a reinforced CS+ (4000 Hz) and two GSs (2000 and 8000 Hz) that were of greater frequency difference from the previously reinforced CS+ but still fell within the octave interval employed in this study; the purpose of the follow-up experiment was to better identify the point of differentiation at which generalization is evident. The second experiment further delineated whether generalization was driven by the effects of CS+/GS adjacency vs. difference in tone frequency. In the follow-up Experiment 2, there was no generalization between the previously reinforced CS+ and these GSs. These data suggest that smaller units of differentiation should be used as GS when employing auditory pure tones to ensure that the resulting gradient is of sufficient sensitivity to detect alterations in anxious clinical populations. In the present study, octaves were selected as units of differentiation based on our previous work on auditory fear conditioning (Norrholm et al., 2011). CSs presented at octave intervals represented salient auditory cues that participants could readily detect and that fell within the range of human experience. The data presented here suggest that future investigations using auditory GSs should use absolute frequency increments (Hz) as units of differentiation.

Lissek (2012) recently discussed fear generalization in terms of the role of hippocampal processing. According to this schema, the presentation of a non-reinforced CS-, following successful fear conditioning to a reinforced CS+, triggers the thalamus to send this sensory information to higher cortical areas involved in sensory processing at which point cortical representations of the CS- are activated (Jarrell et al., 1987; Teich et al., 1988; Lissek, 2012). The hippocampus is then thought to undergo a schematic match, or an appraisal of same vs. different features, between cortical appraisals of the previously reinforced CS+ and the newly encountered CS-. If there is a significant overlap between features of the CS+ and CS-, it is believed that a pattern completion occurs in the hippocampus in which the common features shared by the CS+ and CS- activate a pattern of neural activity originally linked to the CS+ driven fear memory; this activation includes elements of fear neural circuitry including the amygdala and anterior insula. The result of this activation is the generation of a fear response to the newly encountered CS- and generalization of fear at the behavioral level. If there is little or no overlap between the features of the CS+ and CS-, it is believed that a pattern separation occurs at the hippocampal level and the subsequent activation of a medial prefrontal cortical inhibitory signal that attenuates the amygdala-based fear response. The result of this inhibition is the lack of a fear response to the CS- and the absence of fear generalization. Further, the previously discussed fMRI studies from Dunsmoor et al. specifically showed aversion learning modulates activity within category-selective (e.g., animate vs. inanimate) cortex and amygdala, which appears to be modulated by the coupling of the hippocampus to the amygdala during early acquisition (Dunsmoor and LaBar, 2013).

The results of the present study can be interpreted according to the latter models. In Experiment 1, the GSs that were adjacent to the previously reinforced CS+ were 250 and 500 Hz different from the CS+ based on the employed octave interval scale. It appears as though these tones were similar enough to the CS+ to elicit fear-potentiated startle responses of similar magnitude to the CS+ during the Generalization Test. In Experiment 2, there was no observed generalization between the original CS+ (4000 Hz) and the two GSs (2000 and 8000 Hz) that were one unit of differentiation away from the CS+ on the octave interval continuum. The difference in raw frequency at this end of the octave interval scale appears to be such that there was little or no shared similarity between the GSs and CS+ and thus no observed generalization.

Several factors have been identified from previous work as having a significant influence on stimulus generalization. For example, early work demonstrated that fear conditioning to a single CS produces greater generalization than differential fear conditioning tasks (i.e., include both a reinforced CS+ and a non-reinforced CS-; (Jenkins and Harrison, 1960)). Additionally, generalization is influenced by context (Vervliet et al., 2013), and the presence of explicit verbal instructions (Vervliet et al., 2010).

The present study, in a manner similar to recent investigations of stimulus generalization in humans, used GSs that differed along a single stimulus dimension (e.g., frequency). Unlike these more recent reports, this is the first study to employ auditory stimuli, which may provide more robust fear conditioning and better discrimination (Norrholm et al., 2011). Most of the emerging work in this area has used visual cues that most often differ in size (Lissek et al., 2008, 2010; Greenberg et al., 2013b), color wavelength (Dunsmoor and LaBar, 2013), or faces that morphed from neutral to fearful expression (Dunsmoor et al., 2011), or from one individual to another (Glenn et al., 2012; Britton et al., 2013). In the majority of the studies, including the present one, the stimuli are neutral cues prior to conditioning. Nonetheless, one cannot discount the use of more fear-relevant, anxiety disorder specific GSs when examining generalization differences in psychiatrically healthy vs. clinically anxious individuals. The main issue with fear-relevant stimuli (such as morphing from fearful to neutral expressions), presents a confound between generalization of conditioned responses to the US and unconditioned responses to fearful stimuli. Further, eye injuries are prevalent in service members; specifically, during an 11-year surveillance period spanning 2000–2010, there were 186,555 eye injuries diagnosed in fixed medical facilities and between 2005–2010, 8,323 eye injuries were reported from deployed medical treatment facilities (Hilber, 2011). Understanding basic metrics of fear conditioning paradigms across different modalities will enable inclusion of a large subset of a population at an increased risk for PTSD.

### Limitations

There are several limitations in the current study that must be noted. For example, the same context was used for all experimental sessions (Fear Acquisition, Re-training, and Generalization Test) in the current study. During the Generalization Test, there was significant fear-potentiated startle to the CS- used during Fear Acquisition. This appears to be due to elements of context conditioning. Fear potentiated startle studies in humans typically show some conditioned fear to the CS- and this has been observed during “safe” conditions (e.g., extinction; see Norrholm et al., 2006). This is also partially explained by participant expectations during the Generalization Test. This test occurred 10 min after the Re-training session and may have been perceived as an extension of the Re-training session. In addition, human subjects have also expressed an expectation of reversal learning (switching the reinforcement contingency of the CS+ and CS-; (Norrholm et al., 2008); an expectation that may have influenced their responses to the CS- during the Generalization Test.

In the present study, there was a dissociation between fear-potentiated startle responses and US expectancy ratings during the Generalization Test. This is similar to a dissociation observed in our previous work when assessing the return of fear through spontaneous recovery or reinstatement (Norrholm et al., 2008). It is possible that this dissociation reflects that the underlying neural circuitry for startle and expectancy responses is likely different, in that US expectancy is a metric of cognitive awareness of experimental contingencies, while fear-potentiated startle is a metric of amygdala activity (Davis et al., 1993). For example, when examining startle data, but not contingency awareness, PTSD patients show a deficit in inhibiting fear responses (Jovanovic et al., 2012). These results highlight the robustness of peripheral physiological measures in capturing subcortical activation.

Interestingly, discrimination learning has been shown to effect perceptual ratings on the color of the CS+ representing a gradient shift; specifically, the group that was conditioned with a blue CS- rated an ambiguous blue-green CS+ as more green and vice versa (Dunsmoor and LaBar, 2013). Fear conditioning has been previously shown to effect discrimination and generalization of low-level sensory information such an auditory cues (Resnik et al., 2011). The present experiment provides important clinical advances as well as insight into understanding the mechanisms underlying generalization of different trauma-related sensory processes. In the current investigation, we find that the generalization of conditioned fear responses is limited to one unit of difference when the tone frequency of the GS is increased. An important follow-up study will address whether generalization is limited in the same manner when employing GS tones with decreased tone frequency.

## Conclusions

The results of the present study suggest that investigators must critically evaluate their selection of GSs and their differentiation when developing a generalization platform for clinical samples; the incremental differences between the GSs will dictate the presence or absence of floor and ceiling effects which can, in turn, affect the sensitivity of detecting over-generalization in anxious populations.

## Financial Disclosures

Drs. Norrholm, Jovanovic, and Bosshardt report no financial disclosures. Ms. Briscione, Mr. Anderson, Mr. Kwon, and Mr. Warren report no financial disclosures. Dr. Bradley receives grant support or has received awards from the VA Merit Award Program, the American Foundation for Suicide Prevention, and the American Psychoanalytic Association Psychoanalytic Research Fund.

## Conflict of interest statement

The authors declare that the research was conducted in the absence of any commercial or financial relationships that could be construed as a potential conflict of interest.
